# Use of medical marijuana in cystic fibrosis patients

**DOI:** 10.1186/s12906-020-03116-x

**Published:** 2020-10-27

**Authors:** Michael J. Stephen, Jared Chowdhury, Luis Arzeno Tejada, Robert Zanni, Denis Hadjiliadis

**Affiliations:** 1 Jefferson University, 834 Walnut St, Suite 650, Philadelphia, PA 19107 USA; 2 Drexel University, 245 N 15th St, 6th Floor, Philadelphia, PA 19102 USA; 3 Morristown Medical Center, 435 South Street, Suite 310, Morristown, NJ 07960 USA; 4 Robert Wood Johnson Barnabas Health, Unterberg Children’s Hospital at Monmouth Medical Center, 279 Third Ave Suite 604, Long Branch, New Jersey, 07740 USA; 5 Hospital of the University of Pennsylvania, 3400 Spruce St, Philadelphia, PA 19104 USA

**Keywords:** Cystic fibrosis, Medical marijuana, Alternative medicine

## Abstract

**Background:**

The usage and attitudes towards medical marijuana in Cystic Fibrosis (CF) patients is unknown. Through the use of a survey we aim to clarify rates and reasons for use.

**Methods:**

An anonymous survey was sent out to six centers in the Mid-Atlantic region of the United States. Use of and reason for medical marijuana was assessed, along with attitudes of the perceived utility of medical marijuana.

**Results:**

A total of 637 surveys were sent out, and 193 surveys were returned (30.3% return rate). Three did not give consent, and one was empty, for a total of 189 completed surveys. 31 subjects (16.5%) reported having used marijuana for medical purposes in their lifetime, with 29 (15.4%) of these in the past year. The most used forms were edible and vaporized. The most common indications for usage were pain and stress. 28 out of 31 found marijuana to be a great deal effective for their symptoms. 21 of the 31 rated marijuana very important or important to their health. There were two reported side effects, both mild. Of 156 subjects who responded to the question if they would be interested in medical marijuana if available, 72 (46.2%) replied yes.

**Conclusion:**

The use of marijuana for medical reasons was 15.4% in the past year in this sample CF population, although more expressed interest if it was available through prescription. Side effects were rare. CF physicians are going to have to familiarize themselves with advantages and disadvantages of medical marijuana as there is a great deal of interest within the community, and legalization becomes more common.

**Supplementary information:**

**Supplementary information** accompanies this paper at 10.1186/s12906-020-03116-x.

## Background

The availability of and belief in medical marijuana have recently increased dramatically in the United States. Today, 33 states and the District of Columbia have laws permitting its use, giving 63% of the population access [1]. The evidence for the utility of medical marijuana, however, is limited to moderate-quality evidence for chronic pain and spasticity and low-quality evidence for Tourette syndrome, sleep disorders, weight gain in HIV infection and nausea and vomiting due to chemotherapy [2].

Cystic fibrosis (CF) is a genetic disorder most commonly diagnosed at birth. The lungs are the cause of mortality in 90% of patients, but morbidity from chronic pain, depression, and anxiety are becoming more common as lifespan has recently increased [3]. Treatment of CF with marijuana is complicated by the fact that marijuana is often smoked or vaporised, and inhalation from any source by somebody with a chronic lung disease is discouraged. There is no evidence that alternative delivery systems such as vaporisation are safer than smoking [4]. Drug interactions, especially with new CF transmembrane modulator therapies, are areas of concern as the chemically active components of marijuana, cannabinoids, have the potential to induce the liver enzyme CYP1A2 [5].

The current rates and reasons for use of medical marijuana are unknown in the CF population. Only two studies assessing usage rates specifically in CF could be identified in PubMed, both older. The first, published in 1987 by Stern et al., surveyed 173 CF patients and found a 20% marijuana-usage rate [6].

The other study, published in 1998 by Britto et al., showed a 9.7% prevalence in the 115 CF patients surveyed [7]. This study is an attempt to assess current usage rates and to discern the intake route and reasons for medical marijuana usage in a CF population.

## Methods

An electronic survey entitled ‘An Anonymous Survey of Alternative and Complimentary Therapies in Cystic Fibrosis’ was sent via email to patients 16 and older in six CF centres in New Jersey and Pennsylvania. Institutional Review Board approval was obtained at all sites, and the survey was earlier tested on medical residents. The survey was without compensation. Data were collected from March 2017 through February 2018, during which period there were no legal medical marijuana laws for CF patients in either Pennsylvania or New Jersey. Electronic consent outlined the study length, principle investigator and purpose of the survey. Medical marijuana questions were one part of an overall assessment of the use of alternative medicines. Forty-one questions were asked, all on a single page. RedCap, a secure HIPAA compliant data collection tool, was used to collect and store data.

The data were analyzed using SAS version 9.4 (SAS Institute Inc., Cary, NC). Wilcoxon two-sample tests were used to determine whether age group, lung health, or overall health influenced current use of medical marijuana or the use of medical marijuana if it were made legal. A continuity-adjusted chi-squared test was used to determine if gender impacted current or future use of medical marijuana.

## Results

Surveys were sent to 637 subjects, with a total return of 193 surveys, for a return rate of 30.3%. Three subjects refused consent, and one subject left the survey completely blank, leaving 189 surveys for analysis. Baseline characteristics, which overall appear to reflect an average adult CF population, are shown in Table 1. Not all questions were answered by all subjects, so the number of responses is slightly variable by question.

**Table 1:** Baseline demographics and characteristics (N=Number of Responses)

Female, N (%) *N* = 187	112 (59.9)
Age groups, N (%) *N* = 188	
12–20	19 (10.1)
21–35	103 (54.8)
> 35	66 (35.1)
Pancreatic insufficiency, yes (%) *N* = 189	150 (79.4)
Lung health perception, N (%) N = 188	
Excellent	34 (18.1)
Good	84 (44.7)
Fair	55 (29.3)
Poor	15 (8.0)
Overall health perception, N (%) N = 189	
Excellent	30 (15.9)
Good	118 (62.4)
Fair	38 (20.1)
Poor	3 (1.6)

Of the 189 survey completions, 31 subjects (16.5%) reported having used marijuana for medical reasons, and 29 subjects (15.4%) reported having done so in the past 12 months. The route of administration used by subjects was diverse, with 90% reporting edible use, 48% vaporised, 38% smoked, 25% oil, 6% tea, and 6% topical. Edible was the preferred method of 48% of the subjects, while vaporized was preferred by 32%, smoked by 13%, and oil by 6%. Reasons for use are listed in Table 2.

**Table 2:** Reason for using medical marijuana (*N* = 31)

	Number	Percent
Relaxation	25	80.7
Pain	19	61.3
Appetite stimulant	17	54.8
Insomnia	16	51.6
Nausea	13	41.9
Depression	12	38.7
Dyspnoea relief	11	35.5
Migraines	10	32.2
Arthritis	8	25.8

Of the 31 subjects who had used marijuana for medical issues, 28 (90.3%) stated that it was highly effective for their symptoms, with one subject each indicating that it was somewhat, only a little, or not at all effective. Of this same group of 31, 21 thought medical marijuana was either important or very important to their health, while six stated that it was somewhat important and four said that it was not at all important. Two subjects of the 31 reported side effects, with one citing drowsiness and the other reporting fatigue, dizziness, and cold hands and feet.

In response to whether they would be interested in trying medical marijuana if it were available, 72 of the 156 subjects who answered the question responded yes (46%). A text response box was provided for giving a reason as to why they would want to try medical marijuana. In the 44 responses from those who had not tried marijuana previously, 10 reported that they would do so for anxiety, nine for musculoskeletal pain, six for appetite stimulation, four for improved breathing, three for sleep aid and one each for coughing, Parkinson’s tremor, bipolar disorder and to expand consciousness.

The Wilcoxon two-sample test used to determine whether age, lung health, or overall health influenced current or future use of medical marijuana did not achieve significance for any variable. There was a trend for current use among younger subjects, which achieved a *p*-value of 0.062. The continuity-adjusted chi-squared test also showed no significance in the analysis of gender on the current or possible future use of medical marijuana.

## Discussion

Cystic fibrosis is a chronic lung disease that can commonly cause pain, anxiety, depression and insomnia as well as chronic dyspnoea. One study demonstrated that 32.6% of CF patients regularly experience intense to severe pain, while the average rates in adults with CF of anxiety and depression are 21.8 and 27.2% respectively [3, 8]. Medical marijuana has been proposed as a possible treatment for these conditions.

No study has ever documented the usage rates of marijuana specifically for medical use in the CF population. Two prior studies did evaluate the use of recreational marijuana, with a rate of 20% in the study by Stern et al. in 1987, while in 1998 Britto found a rate of 9.7% [6, 7]. In this study, we found a rate of medical marijuana use of 15.4% in the year prior. The reasons for usage in this CF population were varied, with anxiety, musculoskeletal pain and appetite stimulation the most common. Those who used it overwhelmingly found it to be very effective, and adverse events were rare although controlled studies are lacking.

The incidence of medical marijuana use in CF has the potential to increase as this survey was done prior to a legal indication for CF in the states of residence of those surveyed. As reported in this study, 46% of subjects indicated they would be interested in trying marijuana if it were legally available. This would be a substantial rise from the current 15.4% who have used it in the past year.

Use of marijuana has been overall increasing in the U.S. recently, and current rates in the general population mirror usage in this CF population. In a poll conducted in 2018, 14.6% of adults in the U.S. had used marijuana in the past year [9]. In a 2012—2013 poll, the rate of adults in the U.S. who had used marijuana in the past year was 9.5%, while in 2001–2002 the rate was 4.1% [10].

In comparison to CF and the general usage frequency in the U.S. of about 15%, other disease-specific studies have shown variable, but generally higher, rates. One study in the U.S. analysed utilisation in orthopaedic surgery patients, with 34% of patients using marijuana out of a total of 275 completed surveys [11]. Another study in the U.S., analysing a population with gynaecological cancer, showed a rate of 26.7% of 225 surveys analzyed [12]. A 2004 Canadian study found that 28.8% of 104 patients with HIV reported use [13]. In multiple sclerosis, a Spanish study of 175 patients showed a rate of 17.1%, while in the U.K. in 254 surveys the rate was 30% [14, 15]. A large 2019 study demonstrated a higher usage rate of medical marijuana in younger patients, a trend that was also seen in this study [16].

It is possible that the somewhat lower rates seen in our survey compared to other chronic diseases is related to the presence of lung disease, which creates a perceived barrier to either smoking or vaporisation. The cigarette smoking rate in the CF population is 2.1%, much lower than 16.9% in 2017 in the general population [3]. This perceived barrier would potentially disappear with legalized medical marijuana as alternative regulated forms like tinctures and creams would be available.

The use of marijuana does not come without potential for side effects, both short and long term. In a 2015 meta-analysis of 79 medical marijuana studies, overall as well as serious adverse events were greater in the marijuana group [2]. Common short-term adverse events were dizziness, dry mouth, nausea, fatigue, loss of balance and hallucination. A 2010 meta-analysis noted a consistent association between marijuana use and psychotic episodes [17]. Neurocognitive issues, motor vehicle accidents and emergency department visits may also be increased with marijuana use [9]. Deleterious lung effects documented include an increased prevalence of chronic cough, sputum production, wheezing, shortness of breath as well as episodes of acute bronchitis [18]. This lung morbidity could be more deleterious in the CF population which already has underlying pulmonary disease.

Given this, physicians will need to discuss marijuana use with their CF patients and counsel them on the potential for side effects. As importantly, the route of use should be discussed. In this study, vaporisation or smoking marijuana was the preferred delivery for 45% of subjects. In light of their pre-existing lung disease as well as the recent crisis of severe disease with vaporisation, inhalation of marijuana from any delivery system should be discouraged [19].

A limitation of the study was a somewhat low survey return rate, which could imply bias, but overall the cohort was broad based, with 79% reporting pancreatic insufficiency and 74% perceiving either fair or good lung function, both of which are typical of an adult CF population. The data do capture what a substantial segment of CF patients think about medical marijuana. Questions on medical marijuana use were also part of a larger questionnaire on alternative medicine use, so the results would not have been biased by those specifically interested in the subject.

## Conclusion

Compared to the current usage rate in the general U.S. population, the utilisation of marijuana for medicinal purposes was comparable in this specific CF cohort. With increased availability and a patient population that is expected to live longer, usage may increase. Based on this study, CF physicians are going to have to begin incorporating discussions about potential usage of medical marijuana in their patient panels, and concerns about side effects as well as the potential for toxicity with inhalation should be part of that discussion.

## Supplementary information

**Additional file 1.**

## Data Availability

The entire data set is kept in RedCap, a secure online electronic database which is password protected. The datasets used and/or analysed during the current study are available from the corresponding author on reasonable request.

## Abbreviation

CF: Cystic Fibrosis
