# Supplementary material for: Use of medical marijuana in cystic fibrosis patients
Source: BMC Complement Med Ther. 2020 Oct 27;20:323. doi: 10.1186/s12906-020-03116-x (PMC7590463; doi:10.1186/s12906-020-03116-x)
Supplement: Supplementary file 1 — Additional file 1. [file 12906_2020_3116_MOESM1_ESM.pdf]

REDCap

Anonymous Survey of Alternative and Complimentary

You are getting this link and survey to help assess the Cystic Fibrosis community the use of alternative therapies. This is a completely anonymous study. Please feel free to skip any questions including those that you perceive to be intrusive. Your participation is completely voluntary and is not being tracked by your home institution. The survey will be going to a database, and Michael J. Stephen MD at Drexel University is the principle investigator. It will take approximately twenty minutes to complete. Please review the below consent form prior to beginning the survey. Thank you for your consideration.

Drexel University
Institutional Review Board

Electronic Consenting Script

ELECTRONIC CONSENTING
COMMUNICATION TO PROMOTE COMPREHENSION

We are asking you to be in a research study.
You do not have to be in the study, participation is completely voluntary.
If you say yes, please know that you can quit the study at any time.
Please take as much time as you need to make your choice. Call or e-mail us if you need more information.
Your medical or observational care will not change in any way if you say no.
Title of Protocol: "Survey of Alternative Medicine Use in Cystic Fibrosis Patients"

Why are you doing this research study?

The use of alternative medicine is utilized by many patients with cystic fibrosis, and we would like to quantify this.

What happens if I say yes, I want to be in the study?

You will proceed to fill out this anonymous survey, after which no further data will be collected.

How long will the study take?

The survey will take approximately 20 minutes to complete.

What am I being asked to do?

You are being asked to provide your use of various alternative medicines, and if you feel this medicines are helpful.

What happens if I say no, I do not want to be in the study?

Nothing further needs to be done, and you would simply not fill out the survey.

What happens if I say yes, but change my mind later?

If you change your mind while you are filling out the survey, you simply need to close it without submitting, and no data will be transmitted. Unfortunately, since the survey is anonymous, if you submit the results of the survey there is no way to then take it out of the database as we do not know whose survey belongs to whom.

Who will see my answers or my personal information?

We will see you answers, but will not know who the data came from. No personal information is being collected where we could identify you.

Will it cost me anything to be in the study?

No

Will being in this study help me in any way?

Not directly, no.

Will I be paid for my time?

No payment is being offered.

Is there any way being in this study could be bad for me?

No, we do not believe so. However, if you feel any questions are intrusive, please feel free to skip them, or not submit the survey.

What if I have questions?

For any questions, please feel free to reach out to the study principle investigator, Dr. Michael J Stephen at michael.stephen@drexelmed.edu. Also feel free to discuss with the cystic fibrosis director at your site.

What should I do if I want to be in the study?

Please proceed with filling out the survey and hit submit at the end.

By indicating that you have read and understood the consent form, you are saying:
• You agree to be in the study.
• We talked with you about the information in this document and answered all your questions.

You know that:
• You can skip any question you do not want to answer.
• You can stop answering our questions at any time and nothing will happen to you.
• You can call the office in charge of research at (215) 762-3944 if you have any questions about the study or about your rights.

What else do I need to know?

Authorization to Use and Disclose Protected Health Information
Federal law provides additional protections of your personal information that are described here.

A. Individually Identifiable Health Information that will be Collected.
The following personal health information about you will be collected and used during the research study and may be given out to others:
• Information learned during surveys, as specifically relates to use of alternative medicine you use to help with your cystic fibrosis.
A. Who Will See and Use Your Health Information within Drexel University
The researcher and other authorized individuals involved in the research study at Drexel University will see your anonymous survey results during and may give out your anonymous survey results during the research study. These include the researcher and the research staff, the institutional review board and their staff, legal counsel, research office and compliance staff, officers of the organization and other people who need to see the information in order to conduct the research study or make sure it is being done properly. Your anonymous survey results may be disclosed or transmitted electronically.

Did you read and agree to the above electronic consent document?

Yes

No

Please complete the following questions to the best of your abilities:

Who is filling out the Survey?

Age Category of CF Subject:

Gender

Where do you primarily get your care?

Do you use Pancreatic Enzymes?

Overall Lung Function

Overall Health

Please list all of the medications you are currently taking:

Allergies to medications?

Have you EVER seen a provider or practitioner of any of the following for your own health?

Acupuncture?

Aromatherapy

Ayurveda

Biofeedback

Chelation Therapy

Chiropractic Care

Energy Healing

Therapy/Reiki

Yoga (all forms included)

Pilates

Hypnosis

Massage

Naturopathy

Glutamine

Non-CF Vitamins

Selenium

Curcumin

Soy Supplements

Protein Shakes

DURING THE PAST 12 MONTHS, did you see a practitioner for Protein Shakes?

DURING THE PAST 12 MONTHS, how many times did you see a practitioner for Protein Shakes?

DURING THE PAST 12 MONTHS, did you let any CONVENTIONAL medical professionals know about your use of Protein Shakes?

DURING THE PAST 12 MONTHS, how important was your use of Protein Shakes in maintaining your health and well-being? Would you say?

DHA (Docosahexaenoic Acid)

EFA or any other fatty acid supplements

Folk Medicine (Curanderismo, Native America healing)

Have you EVER used natural herbs for you own health or treatment? (for example, ginger, echinacea, or black cohosh - including teas, tinctures and pills)

People who use homeopathy to treat health problems take small pills or drops that are placed under the tongue. These pills or drops are often prescribed by practitioners of homeopathy. Have you EVER used homeopathic treatment for you own health?

Have you EVER used any of the following relaxation techniques for your own health or treatment? (Please Check all that Apply)

Did you use (this/these) relaxation technique(s) to treat a specific health problem or conditions?

For what health problems or conditions did you use (this/these/ relaxation technique(s)?

How much do you think (this/these) relaxation technique(s) helped your health problems or conditions?

DURING THE PAST 12 MONTHS, how important was your use of (this/these) relaxation technique(s) in maintaining your health and well being?

DURING THE PAST 12 MONTHS, did you see a practitioner for relaxation techniques?

DURING THE PAST 12 MONTHS, did you let any of these CONVENTIONAL medical professionals know about your use of (this/these) relaxation techniques?

Have you EVER prayed specifically for the purpose of your OWN health?

Have you EVER used marijuana for treatment of your health problems or conditions?

DURING THE PAST 12 MONTHS, did you use any form of marijuana for treatment of your health problems or conditions?

What form of marijuana did you use? (check all that apply)

Which form of marijuana did you find to have the most beneficial effect on your health?

For what reasons did you used the marijuana? (check all that apply)

How effective was marijuana in relieving your symptoms?

DURING THE PAST 12 MONTHS, how important was your use of marijuana in maintaining your health and well-being?

Did you suffer any side effects from marijuana use?

If medicinal marijuana was available, would you be interested to see if it can help with your condition?

Why?

In the past 12 months have you ever used marinol (dronabinol)?

In regards to any alternative or complementary medicine, did you preferentially use these over the medications prescribed by your Cystic Fibrosis Physician?

What was the reason you preferred alternative or Complemenatry medicine? (check all that apply)

Can you please tell us about any other experiences you have had with any other alternative therapy or medicine (not listed above) used for CF management? Please list the therapy or medicine, how performed or route of delivery, how long you used it, and the benefits of the therapy or medicine.
